# Muscle Synergies in Parkinson’s Disease

**DOI:** 10.3390/s20113209

**Published:** 2020-06-05

**Authors:** Ilaria Mileti, Alessandro Zampogna, Alessandro Santuz, Francesco Asci, Zaccaria Del Prete, Adamantios Arampatzis, Eduardo Palermo, Antonio Suppa

**Affiliations:** 1 Department of Mechanical and Aerospace Engineering, Sapienza University of Rome, 00184 Rome, Italy; ilaria.mileti@uniroma1.it (I.M.); zaccaria.delprete@uniroma1.it (Z.D.P.); eduardo.palermo@uniroma1.it (E.P.); 2 Department of Human Neurosciences, Sapienza University of Rome, 00185 Rome, Italy; alessandro.zampogna@uniroma1.it (A.Z.); francesco.asci@uniroma1.it (F.A.); 3 Department of Training and Movement Sciences, Humboldt-Universität zu Berlin, 10115 Berlin, Germany; alessandro.santuz@hu-berlin.de (A.S.); a.arampatzis@hu-berlin.de (A.A.); 4 Berlin School of Movement Science, Humboldt-Universität zu Berlin, 10115 Berlin, Germany; 5 Atlantic Mobility Action Project, Brain Repair Centre, Department of Medical Neuroscience, Dalhousie University, Halifax, NS B3H 4R2, Canada; 6 IRCCS Neuromed, 86077 Pozzilli (IS), Italy

**Keywords:** Parkinson’s disease, muscle synergies, motor modules, motor primitives, electromyography, balance, locomotion, gait

## Abstract

Over the last two decades, experimental studies in humans and other vertebrates have increasingly used muscle synergy analysis as a computational tool to examine the physiological basis of motor control. The theoretical background of muscle synergies is based on the potential ability of the motor system to coordinate muscles groups as a single unit, thus reducing high-dimensional data to low-dimensional elements. Muscle synergy analysis may represent a new framework to examine the pathophysiological basis of specific motor symptoms in Parkinson’s disease (PD), including balance and gait disorders that are often unresponsive to treatment. The precise mechanisms contributing to these motor symptoms in PD remain largely unknown. A better understanding of the pathophysiology of balance and gait disorders in PD is necessary to develop new therapeutic strategies. This narrative review discusses muscle synergies in the evaluation of motor symptoms in PD. We first discuss the theoretical background and computational methods for muscle synergy extraction from physiological data. We then critically examine studies assessing muscle synergies in PD during different motor tasks including balance, gait and upper limb movements. Finally, we speculate about the prospects and challenges of muscle synergy analysis in order to promote future research protocols in PD.

## 1. Introduction

Parkinson’s disease (PD) globally affects 6.2 million people, representing the second most common neurodegenerative disorder after Alzheimer’s disease [1]. By 2040, it is estimated that up to 17 million people worldwide will suffer from PD, thus representing a “Parkinson pandemic” [2]. PD is a neurological disorder characterized by dopaminergic neuron depletion in the midbrain structure called the substantia nigra pars compacta and associated with intracellular and extracellular inclusions of a misfolded protein, α-synuclein [3]. The hallmark motor symptoms of PD consist of bradykinesia (i.e., movement slowness and reduction), rest tremor, and rigidity (i.e., increased muscle tone with “lead-pipe” resistance to passive movement), which are often associated with postural instability in more advanced disease stages [4]. l-dopa and other drugs acting on dopaminergic transmission are the most effective medical treatment for motor symptoms in PD patients [5,6]. Nevertheless, therapeutic management of the advanced disease stages is rather challenging due to complications related to the chronic intake of drugs, such as l-dopa-induced dyskinesia [5]. Accordingly, clinical strategies to manage advanced disease stages include pharmacological treatments based on the continuous infusion of l-dopa/carbidopa intestinal gel or apomorphine, and non-pharmacological approaches based on deep brain stimulation (DBS) of the subthalamic nucleus and the globus pallidus pars interna [5,7]. 

A major challenge in the clinical management of PD patients concerns the occurrence of axial motor symptoms, including balance and gait disorders [8]. Postural instability and gait disturbances, such as the paroxysmal interruption of locomotion (i.e., freezing of gait) [9], severely affect patient quality of life by reducing individual independency and increasing the risk of falls and injuries [8]. These symptoms are largely refractory to pharmacological and non-pharmacological treatment [5]. Furthermore, since axial symptoms strongly impact the natural history of PD, they are crucial to clinically stage the disease, according to the Hoehn and Yahr scale (H&Y) [10]. Despite considerable scientific efforts to clarify the mechanisms leading to these symptoms [11,12,13], the pathophysiology underlying postural instability and gait disorders in PD remains largely unclear [11,14]. Accordingly, new experimental approaches are required to investigate motor control in patients with PD during balance maintenance and gait to implement current therapeutic strategies for these symptoms.

Over the last 30 years, innovative computational methods have led to the alternative approach of analysing muscle synergies in order to study human motor control [15,16]. Muscle synergy is the coordinated recruitment of a set of muscles, whose activation is strictly balanced in amplitude and timing with several possible arrangements, to perform purposeful movements [16]. According to the modular organization of motor control, muscle synergies have been proposed as a physiological model adopted by the central nervous system (CNS) to flexibly perform movements by minimizing the neural processing of motor output by producing various muscle patterns [16]. Muscles synergies have been previously demonstrated in animals [16,17,18,19,20,21,22] and healthy humans [23,24]. Moreover, they have also been examined in patients suffering from neurological disorders, including PD [23].

This narrative review aims to examine previous findings concerning the assessment of motor symptoms in PD patients through the analysis of muscle synergies. Accordingly, we first discuss the problem of motor control and the theoretical model of muscle synergies. We then summarise computational methods currently used to estimate muscle synergies. We critically examine previous studies addressing this topic in PD. Finally, we discuss technical and clinical challenges and speculate about the prospects of muscle synergies assessment in PD patients. 

## 2. Muscle Synergies: Theoretical Background

### 2.1. The Modularity of Movement and Muscle Synergies

The motor system of vertebrates presents countless degrees of freedom, intended as the number of ways the system can independently vary, due to multiple anatomical (e.g., muscles and joints), kinematic (e.g., trajectories, accelerations, and velocities) and neurophysiological (e.g., motoneurons and neuromuscular junctions) variables that determine the execution of movements [25]. Thus, different combinations of anatomical, kinematic and neurophysiological elements can be used to achieve a specific and purposeful movement, resulting in many possible ways to perform the same motor task. Accordingly, the redundancy of the motor system and the problem of motor equivalence (i.e., multiple solutions to perform the same movement) leads to the question of how the CNS manages such high-dimensional data and selects one solution from among others [25,26]. Despite the availability of several equivalent motor solutions, movement execution commonly involves stereotyped motor behaviours (e.g., locomotion) [26,27]. This suggests that the CNS may adopt predefined strategies to solve the problem of redundant degrees of freedom and simplify motor control. Accordingly, experimental observations [17,18,19,20,28,29] support the hypothesis that motor control is based on a modular structure, consisting of basic configurations of muscle activations (i.e., synergies) whose variable combination is responsible for several muscle patterns and complex motor behaviours [30]. Hence, through the modularity of movement (i.e., a small number of shared muscle synergies for many movements), the CNS could effectively reduce high-dimensional data to low-dimensional elements by using a few combination coefficients [30].

**Figure 1:**
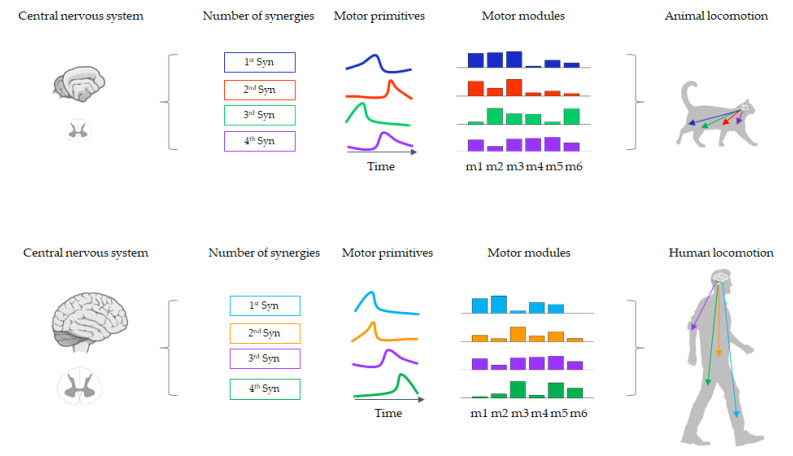
A representative example of the number of synergies, motor primitives and motor modules in animals and humans while walking.

Over the last decades, several theoretical models of modular control have been proposed (e.g., neuromotor synergies, unit burst generators, spinal force fields) [15,29,30,31]. However, new computational approaches and experimental evidence ultimately led to the current concept of muscle synergies [16]. Depending on the specific motor task to be performed, the CNS manages the activation levels (spatial domain) and synchrony (temporal domain) of all muscles included in each synergy [32]. Concerning the temporal domain, in “time-invariant synergies” all muscles within the synergy are synchronously activated (i.e., without muscle activation delays), while in “time-varying synergies” each muscle shows a distinct temporal profile (i.e., reciprocal delay in muscle activation) [33]. By scaling amplitudes and muscle activation delays, the linear combination of a few muscle synergies could allow the CNS to flexibly generate a large number of different muscle patterns [32]. Some muscle synergies are task-specific, while others may be shared between different motor behaviours [34]. Moreover, sensory feedback helps to modulate the recruitment of muscle synergies and adapt motor output to the external environment [22,35] (Figure 1). 

Experimental studies have supported synergistic movement in several motor conditions in cats [19,20], frogs [16,17,36] and mice [22]. One of the most relevant models comes from the study of motor control during postural tasks in cats [19]. Electromyographic (EMG) analysis of cat hindlimb muscles has shown that just four synergies could account for more than 95% of automatic postural responses during multidirectional postural perturbations [19]. These findings have demonstrated that even motor behaviour as complex as dynamic balance control can be reconducted to a small number of coordinative patterns [19]. Similarly, human studies extracted six or fewer muscle synergies during multidirectional support-surface translations by recording 16 back and leg muscles [37]. Beyond postural responses, studies in animals [20,22,38,39] and humans [40,41,42] have examined other motor behaviours, including locomotion and arm movements, through muscle synergy analysis. Concerning locomotion in humans, a specific number of muscle synergies can explain each phase of the gait cycle [43,44]. Four or five synergies are the minimum number required to sufficiently reconstruct the measured EMG activity during human locomotion [43,44]. These synergies also contribute to reactive balance control [45]. Moreover, previous authors have identified a small set of muscle synergies able to reconstruct a large number of arm movements during multidirectional reaching tasks [42].

Although the neural origin of muscle synergies is highly debated [34,46,47], strong evidence from animal studies supports the existence of specific neural controllers along the CNS [34]. By using different stimulation approaches (e.g., microstimulation, N-methyl-D-aspartate iontophoresis, and cutaneous stimulation), several authors have demonstrated modular movement organization in the spinal cords of frogs, rats, and cats [15,16,28,48,49]. The activation of spinal interneuronal networks leads to stereotyped motor patterns of muscles groups, thus unveiling distinct spinal motor modules, including the central pattern generators for locomotion [50]. Experiments that involved transecting the frog neuraxis at different levels above the spinal cord have also identified neural circuits expressing muscle synergies in the brainstem, mainly in the medulla [51]. These experiments have shown that the brainstem and spinal cord express most of the muscle synergies for motor behaviours in vertebrates [51]. Conversely, high-level motor structures, including the primary motor cortex (M1) and other non-primary motor areas such as the supplementary motor cortex and premotor cortex, likely contribute to the activation and coordination of low-level neural structures to properly perform complex motor tasks, by selecting a subset of muscle synergies [34,51]. Accordingly, M1 activity relates to EMG patterns during the performance of different motor tasks in monkeys and cats [38,52,53]. Moreover, an anatomic study in monkeys [54] has demonstrated that M1 includes a rostral, phylogenetically older portion connected with spinal interneurons (“old M1”), which is likely involved in synergy modulation [38,51]. Additionally, a rostral, phylogenetically more recent division of M1 (“new M1”) directly innervates spinal motoneurons and likely activates individual muscles for more agile movements in primates [51,54]. Furthermore, the study of patients suffering from CNS lesions has contributed to the understanding of the neural substrate of muscle synergies in humans. Consistent with animal findings, patients with stroke-related lesions in motor cortical areas have shown a similar minimum number of muscle synergies to explain a specific amount of data variation in EMG signals for voluntary movements of the affected and unaffected arms [55]. Also, the two sets of synergies extracted in the affected and unaffected arms included similar muscle contributions, suggesting the preservation of low-level neural structures in patients with lesions of motor cortical areas [55]. Accordingly, the abnormal coordination of muscle synergies due to disrupted descending motor commands could explain motor performance differences between the affected and unaffected arms [51,55]. Further supporting this hypothesis, lesions of the spinal motor modules and the loss of descending motor pathways may explain muscle synergy changes in patients affected by spinal cord injuries [56,57].

### 2.2. Methods for Muscle Synergy Extraction

As stated above, muscle synergies are usually extracted from EMG signals [16]. The concept behind traditional extraction approaches is to identify common EMG patterns recorded from multiple muscles during any motor activity. A large number of muscle activities can be decomposed or grouped into a lower number of common activation patterns and relevant weights. For instance, during human locomotion, foot plantar flexors are used for propulsion in the second half of the stance phase, when their weight (i.e., “importance”) is highest. However, they are not as important in the early swing phase when their activity, and thus their weight for that pattern, is close to zero [44,58]. Several unsupervised machine learning methods are available that aim to identify which muscles work synergistically by linear decomposition of EMG signals [59,60]. In general, these methods attempt to build a model based on the linear combination of synergies following rules similar to:

(1) $$m\left(t\right)=\overset{r}{\underset{i=1}{\sum }}c_{i}\left(t\right)w_{i}+\varepsilon$$

where **m**(*t*) is a vector containing time-dependent activations of the recorded muscles at a specific time point (or time interval [33]) *t*, *r* is the number of synergies, **w***_i_* is a time-independent vector of weights (henceforth the motor modules), *c_i_*(*t*) is a time-dependent set of coefficients (henceforth the motor primitives) and *ε* is an error [16]. Some of these techniques include principal component analysis (PCA), independent component analysis (ICA, a special case of blind source separation), non-negative matrix factorization (NMF), extreme learning machines (ELM) and the generalized tensor decomposition techniques [16,23,30,59,60,61,62,63,64,65,66,67,68,69,70,71,72,73,74,75,76,77,78,79,80,81,82]. 

While they have been shown to have similar performance [59,70], factorization approaches rely on different assumptions. For instance, PCA assumes that synergies, or the so-called principal components, are orthogonal (i.e., perpendicular). This assumption produces a unique solution for every decomposition [30,82]. If synergies are orthogonal, it follows that they are uncorrelated, allowing for an accurate estimation of how much of the total variance is explained by each synergy. However, for the orthogonality assumption to be satisfied, PCA must allow for negative motor modules [30]. This is a rather counterintuitive feature since muscle activations can be either positive (i.e., when a muscle is active) or null (i.e., when a muscle is at rest), but never negative. A method that overcomes this issue is NMF. As the name implies, NMF constrains the motor modules to be nonnegative, i.e., either positive or null. This condition allows easier interpretation of the outcomes, especially in settings such as clinical environments where qualitative interpretability may be of high value. When considering the NMF method, the linear regression of the R^2^ vs. factorisation rank [62] and the threshold on the reconstruction quality [80] are the main approaches to determine the minimum number of synergies. Conversely, PCA commonly adopts a fixed number of muscle synergies based on the Kaiser criterion [83].

**Figure 2:**
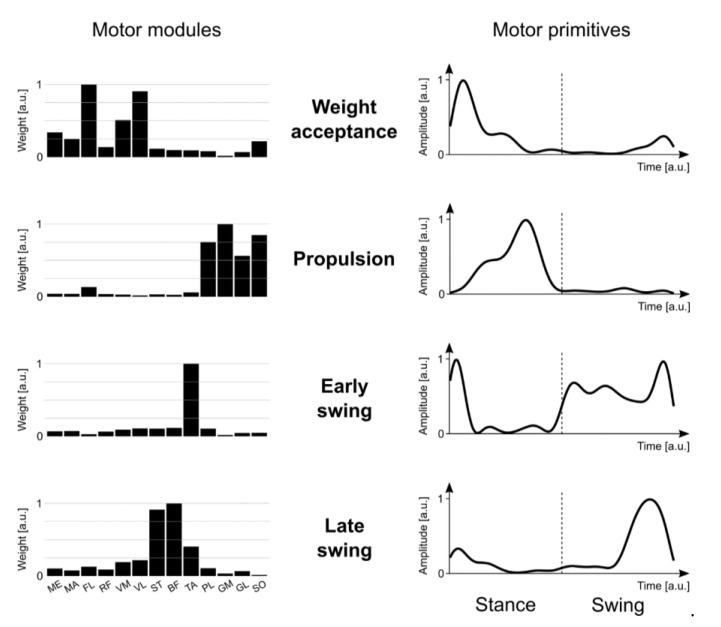
Muscle synergies for human walking. Exemplary motor modules and motor primitives of the four fundamental synergies for human walking extracted via nonnegative matrix factorization (NMF) on a unilateral muscle set. Motor modules are presented on a normalized y-axis base in arbitrary units. For motor primitives, the x-axis full scale represents the averaged gait cycle (with stance and swing normalized to the same amount of points and divided by a vertical dotted line), while the y axis represents the normalized amplitude in arbitrary units. Muscle abbreviations: ME = *gluteus medius*, MA = *gluteus maximus*, FL = *tensor fasciæ latæ*, RF = *rectus femoris*, VM = *vastus medialis*, VL = *vastus lateralis*, ST = *semitendinosus*, BF = *biceps femoris*, TA = *tibialis anterior*, PL = *peroneus longus*, GM = *gastrocnemius medialis*, GL = *gastrocnemius lateralis*, SO = *soleus.*

Figure 2 shows typical NMF output, where non-negative motor modules are represented as bars. A high bar translates to a high contribution (or importance) of that muscle for that specific synergy, while a low bar implies the opposite. However, the advantage of being more physiologically contextualized and understandable for lay readers come at a cost. NMF is obtained with a search algorithm that starts with random values and iteratively separates the synergies via successive iterations [84]. This process produces different, though extremely similar, solutions at every run. Moreover, since the factorized elements are not orthogonal, they are not uncorrelated, notwithstanding their linear independence (i.e., no synergy can be a linear combination of others) [30,82].

Another important consideration on factorization methods is that each of them can be applied in many ways and outcomes can vary depending on the initial data conditions. For instance, several NMF algorithms are based on different mathematical models [63] and an acceptable consistency in results can only be obtained using different criteria, such as those required to stop the iteration process once an adequate number of synergies is found [62,63]. The necessary process of filtering EMG data before factorization is another factor that can influence outcomes. Studies have shown that it is possible to find filtering configurations that reduce the variability of the obtained NMF when calculations are done several times on the same data set [62,78,80]. Moreover, the way initial random values are selected before starting NMF plays a role in the quality of the calculated synergies [64]. Additionally, the number and choice of recorded muscles and gait cycles have been shown to influence factorization [74,75]. Indeed, the number of extracted synergies can change according to the number and type of muscles recorded (e.g., upper and lower limb muscles alone or in combination) [62,74]. Choosing at least 10 superficial muscles and recording at least a few tenths of gait cycles may be the best method to obtain consistent results [74,75]. Direct comparison of studies from different research groups also suffer some difficulties because both motor modules and primitives can undergo arbitrary normalisation procedures. Another important point is that approaches such as PCA, NMF, and ICA only allow for the identification of dependencies between two factors: motor modules (spatial factor) and motor primitives (temporal factor) [67]. When more than two factors are present in the analysis (e.g., when analysing synergy dependence on locomotion speed or motor task), a sophisticated statistical analysis must be custom built due to the low consensus currently in literature [67]. To solve this issue, a generalization of matrix decomposition, i.e., tensor decomposition, has been proposed in recent years [66,67,81]. A matrix is a two-dimensional tensor, but tensors can accommodate more than two dimensions, with the third being the locomotion speed and/or locomotor task, for example. This allows for all factors to be included in the decomposition, thus basing the entire analysis on the same statistics rather than on different approaches (e.g., NMF/PCA/ICA and then analysis of variance, or ANOVA) [66,67,81].

## 3. Literature Search Strategies and Criteria

Two independent researchers (I.M., A.Z.) used Medline, Scopus, PubMed, Web of Science, EMBASE, and the Cochrane Library databases to perform a literature search of studies investigating muscle synergies in PD. The following keywords were used: “Parkinson’s disease” OR “parkinsonian” OR “parkinsonism” AND “electromyography” OR “EMG” OR “factorization” AND “muscle synergies” OR “muscle coordination” OR “motor control” OR “motor module” OR “muscle modes” OR “motor primitives” AND “bradykinesia” OR “rigidity” OR “tremor” OR “balance” OR “postural responses” OR “gait” OR “locomotion” OR “movements”. Hyphens and inverted commas were used to consider all possible keyword combinations. Experimental studies based on EMG recordings that were published from January 2000 to April 2020 were considered for eligibility. Also, references of each matched article were carefully examined so as not to exclude related articles that were not identified in the electronic databases. To avoid terminology confusion, we only included findings from previous studies investigating muscle synergies intended as coordinated groups of muscle activation that are strictly balanced in amplitude and timing with several possible arrangements [32], also called *muscle modes* by some authors [71]. We did not address the topic of synergies as a pathological pattern of muscle activation [85] or as a neural organization of elements for the maintenance of motor stability through the concept of variables “abundance” [86]. Reviews, reports, and articles in languages other than English were also excluded from the literature search. Eligible studies were first collected based on the title and abstract. Afterwards, full texts were evaluated according to the inclusion and exclusion criteria. Extracted data included the demographic and clinical features of PD patients (e.g., sex, age, weight, height, disease duration, clinical phenotype, disease staging and severity, therapy), experimental protocols (e.g., recorded muscles, tasks, synergy extraction) and outcome measures (e.g., muscle synergies and related parameters).

## 4. Muscle Synergies in Parkinson’s Disease

Ten research articles investigated muscle synergies in PD patients [71,83,87,88,89,90,91,92,93,94]. According to the aim of the study, the research articles could be divided into three main groups: (i) six articles investigating muscle synergies during postural tasks with and without external perturbations (balance) [71,83,89,90,91,93], (ii) three articles investigating muscle synergies during walking tasks (locomotion) [87,88,93], and (iii) two articles investigating muscle synergies during upper limb movements (resting tremor and reaching) [92,94]. Three out of the six articles investigating muscle synergies during postural tasks in PD were reported by a single research group [71,83,90]. A different team of researchers conducted two out of three studies examining muscle synergies during walking [87,88]. Lastly, two articles [89,94] re-analysed data already discussed in other manuscripts [71,83,92]. 

Previous studies enrolled PD patients according to different inclusion criteria, including (i) diagnosis of idiopathic PD [71,83,87,88,89,90,91,92,93,94], (ii) absence of clinically overt postural instability [71], (iii) absence of other neurological disorders [89,90] or any other comorbidity possibly affecting motor control, including polyneuropathies or significant visual, vestibular, or musculoskeletal disorders [91], (iv) absence of cognitive impairment [91,92], and (v) ability to independently perform the experimental motor tasks in any state of therapy, including active and inactive deep brain stimulation (respectively DBS-ON and DBS-OFF) [90]. Demographic and anthropometric data considered in most of the studies included: (i) age, (ii) body mass, (iii) height, (iv) sex, (v) disease onset side, and (vi) disease duration. One study also reported the number of years under therapy with DBS [90]. Each study investigating muscle synergies in PD enrolled a mean of 10 patients.

Most studies used the Unified Parkinson’s Disease Rating Scale (UPDRS) part III [71,83,88,89,90,91,92,93] and the H&Y [83,91,92,93] to assess motor symptoms in PD patients. Two studies provided information about patients’ clinical phenotype (i.e., tremor dominant or postural instability/gait difficulty dominant) [92,93]. Only half of the studies [83,91,92,93] specified the disease stage by including patients with early (i.e., H&Y I-II) and mid (i.e., H&Y III) disease stages. Only one study [91] also used the Berg balance scale to examine postural control in PD patients. Some authors evaluated cognitive function through the Mini-Mental State Examination [91,92]. Four studies reported the L-Dopa equivalent daily dose to calculate the total amount of drug intake [71,83,90,92]. 

The experimental tasks used to assess muscle synergy in healthy subjects (HS) and PD patients included several setups based on the motor behaviour to be studied. Balance evaluation employed various motor tasks such as quiet standing [71,83,90], self-triggered postural perturbations [71,83,90], and multidirectional external perturbations [91]. Furthermore, locomotion was analysed by measuring muscle synergies during overground walking [88,93] and walking on a treadmill [87,88]. Only one study [92] investigated muscle synergies in upper limb movements of PD patients during resting tremor and reaching tasks.

All studies investigated muscle synergies in PD patients under dopaminergic therapy (ON therapy), whereas only three studies [83,88,91] examined patients who were not under dopaminergic therapy (OFF therapy). ON therapy referred to 1–3 h after the last drug intake [83,88,91,92] or a self-determined best medical condition [93], whereas OFF therapy required at least 12 h of drug withdrawal [83,88,91]. 

**Figure 3:**
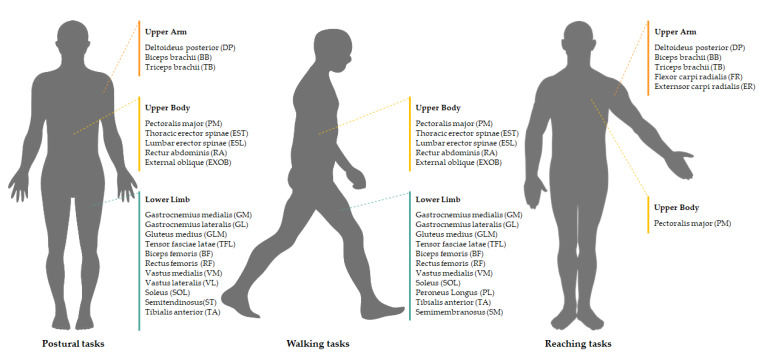
Muscles recorded by surface EMG to analyse muscle synergies in Parkinson’s disease patients during postural, walking and reaching tasks.

All studies recorded EMG signals from a set of 22 muscles of the upper limb, upper body, or lower limb by using surface electrodes. Figure 3 summarises the recorded muscles according to the motor tasks. Six studies extracted muscle synergies by applying the NMF approach combined with the variability accounted for (VAF) (i.e., the correlation coefficient between measured EMG signals and reconstructed EMG signals) [87,88,91,92,93,94], whereas the rest used PCA with factor extraction after Varimax rotation [71,83,89,90]. Major metrics used in the statistical analysis were: (i) number of muscle synergies at the 95% VAF [87,88,91,93]; (ii) amplitude of the individual contribution of each muscle to the muscle-weighting vectors [87,88]; (iii) amplitude and timing of the activation profile [87,88,92]; (iv) %VAF for the individual muscle [87,88]; (v) total %VAF [88,91]; and (vi) total variance of the first four muscle modes [71,83,89,90].

### 4.1. Balance

Concerning balance, Falaki et al. [71] used the PCA technique to assess muscle pattern during self-triggered postural perturbations (i.e., upright voluntary sway, fast sway, and a load release task), showing that in PD patients the first four muscle synergies accounted for a lower variance in muscle activation than in HS. In a further study in PD patients, the same authors [83] also demonstrated that the first four muscle synergies accounted for a higher amount of variance in patients ON as compared to OFF therapy. Conversely, a third study from the same authors found that DBS leaves these measures unchanged in patients with PD [90]. By adopting the NMF approach, Allen et al. [93] demonstrated post-rehabilitative improvement of within- and between-synergy structures (i.e., consistency, distinctness, and generalizability) without changes in the number of synergies during multidirectional translations of the support surface in PD patients. Lastly, Mileti et al. [91] demonstrated that PD patients without clinically overt postural instability use a lower number of synergies than HS to maintain upright stance in response to external postural perturbations around the vertical axis. These measures were unresponsive to L-Dopa and correlated with cognitive and motor function, as reflected by mini-mental state examination scores and the “body bradykinesia” subitem of the UPDRS-III.

### 4.2. Locomotion 

Concerning locomotion, Rodriguez et al. [87] measured EMG activity from eight leg muscles bilaterally during a 10-min walking task on a treadmill, demonstrating that PD patients require fewer synergies for the reconstruction of muscle activation patterns than HS. Moreover, PD patients showed abnormal motor primitives (or activation profiles) both in amplitude and timing as compared with HS, but similar motor modules (or muscle weights). The same authors [88] also demonstrated that dopaminergic therapy does not affect the number, structure, or timing of muscle synergies while walking. Muscle synergies correlated with walking speed during ON therapy, but not with stride time or length [87,88]. Similar to balance evaluation findings, Allen at al. [93] reported a post-rehabilitative improvement in consistency, distinctness, and generalizability in muscle synergies during locomotion, whereas the number of muscle synergies remained unchanged.

### 4.3. Upper Limb Movements 

Regarding upper limb movements, Hu et al. [92] analysed the effects of transcutaneous electrical stimulation of the radial nerve on resting tremor and reaching movements in PD patients. The authors found that this experimental intervention modulates the time profile amplitude of muscle synergies differently during resting tremor and reaching tasks, but does not influence the number of synergies.

The demographic and clinical features of the PD patients included in these studies investigating muscle synergies are summarised in Table 1. Moreover, a detailed description of the methods and findings from these studies is reported in Table 2, excluding the two manuscripts [89,94] that re-analysed data already discussed in other research articles [71,83,92].

## 5. Discussion 

To date, several studies have examined muscle synergies in PD patients to investigate pathophysiological mechanisms underlying motor symptoms and the effect of dopaminergic therapy. To this aim, several authors have used multiple motor tasks primarily involving balance and locomotion to examine muscle synergy in PD.

When comparing PD patients and HS, despite the different experimental approaches used to assess muscle synergies (e.g., muscle sets and data processing techniques), all studies consistently found a similar number of synergies between groups during postural and walking tasks [71,87,91]. Also, Allen et al. [93] demonstrated a similar number of muscle synergies before and after a specific neurorehabilitation protocol in PD patients. Lastly, in addition to balance and locomotion, a further study in PD patients examining upper limb movements (tremor and reaching) also confirmed a comparable number of synergies before and after non-pharmacological intervention based on peripheral nerve electric stimulation [92]. However, the amount of variance in muscle activation that can be explained by a fixed number of synergies differed in PD patients as compared with HS, with some inconsistency. By considering an equal number of muscle synergies, Mileti et al. [91] and Rodriguez et al. [87] both observed higher %VAF values in PD patients as compared with HS. Conversely, Falaki et al. [71] found a lower amount of variance in PD patients than in HS. However, the authors considered higher [87,91] and lower %VAF values [71] as a measure of lower motor performance in PD patients as compared with HS, making it difficult to consistently interpret these findings. There are multiple explanations for the inconsistencies among studies, including differences in patient clinical features, methodological approaches for muscle synergy extraction, and motor tasks used. Moreover, concerning locomotion, walking speed is a further confounding factor to be considered [95]. However, due to the small number of studies investigating this issue in PD and the lack of experimental assessments with muscle synergy analysis in PD animal models, it is currently difficult to reach a definitive conclusion. Despite some inconsistency among studies, some parameters obtained from muscle synergy analysis could distinguish between PD patients and HS. More in detail, when considering a fixed number of synergies, the main differences between PD patients and HS would regard the %VAF and measures of within- and between-synergy structures primarily related to the motor primitives [87,93]. A similar consideration applies when considering the effect of L-Dopa on muscle synergies. Mileti et al. [91] and Roemmich et al. [88] demonstrated similar muscle synergies in PD patients under OFF and ON therapy during postural and locomotion tasks, seemingly consistent with the clinical observation that axial motor symptoms are frequently unresponsive to therapy [8]. In contrast with these findings, Falaki et al. [83] reported a more consistent organization of muscle synergies associated with an increased amount of %VAF during postural tasks in the ON as compared to OFF state in PD patients. Moreover, the same authors [90] found that DBS does not influence postural performance in PD patients, a finding possibly consistent with the poor DBS-related improvement of axial motor symptoms. Findings concerning muscle synergies in PD agree with previous studies in patients affected by other CNS disorders. Indeed, patients with stroke or cerebral palsy have shown heterogeneous changes in muscle synergies based on the lesion extent and location [96,97,98]. For instance, cortical damage in patients with stroke did not affect the internal structure but changed the modulation, of muscle synergies [55]. Conversely, extensive hemispheric strokes or brain injury in cerebral palsy significantly impacted on the number and structure of muscle synergies [97,98]. All these observations support the hypothesis that, in humans, high-level nervous structures coordinate and modulate muscle synergy controllers that are likely localised in the spinal cord and brainstem [34].

Overall, when considering the inconsistencies among studies, several clinical and technical issues should be noted. One of the main clinical concerns regards the sample size and heterogeneity of patient cohorts. All studies on muscle synergies in PD patients included a small number of subjects (10 patients per study on average), thus implying low statistical power. Moreover, these studies mostly showed poor clinical characterization of PD patients. Accordingly, to improve the scientific quality and resolve open clinical questions, future studies should enrol larger numbers of patients. Moreover, strict inclusion criteria should include only patients selected according to the current gold standard diagnostic criteria [4] and exclude those with neurological and other medical comorbidities that may impact motor control. Demographic and anthropometric features of enrolled subjects should always be reported. Furthermore, PD patient assessment should include evaluation of the clinical phenotype (i.e., tremor dominant or postural instability/gait difficulty subtypes), as well as the severity and staging of the disease through standardized tools such as the UPDRS and H&Y. The use of standardized clinical scales is required to accurately select homogeneous cohorts of PD patients affected by a specific clinical phenomenon. Cognitive and psychiatric disturbances, including mood disorders, should also be evaluated through standardized clinical scales such as the Montreal Cognitive Assessment, outcome scales in PD cognition, or the hospital anxiety and depression scale. According to the specific symptoms investigated, additional standardized clinical scales, such as the Berg scale for balance, the new freezing of gait questionnaire for freezing of gait, or the Timed Up and Go test for locomotion, should be adopted. This would allow a better investigation of possible clinical-behavioural correlations that could support the overall interpretation of findings. Lastly, a further crucial point in PD patients concerns the evaluation of L-Dopa effect. Indeed, the effect of PD *per se* can be distinguished from the effect of dopaminergic therapy only by comparing measures in patients OFF and ON therapy. Similarly, when examining the effect of DBS, all measures should be collected in patients under DBS-OFF and DBS-ON conditions, while OFF and ON therapy. 

Several studies have provided methodological recommendations to allow a more comprehensive analysis and guarantee comparability of results [59,74,75,78,80,99,100]. Recommendations reported in previous studies, such as the number and selection of muscles [74], pre-processing techniques [75], filtering processes [62], number of trials [75], or factorization algorithm selection [59,62], have only partially been applied to PD cohorts. Moreover, both reliability and repeatability of EMG factorization play an important role in the correct interpretation of muscle synergies [62,80,95]. Hence, to validate the use of muscle synergies in PD, the repeatability of the synergy structure should be examined by analysing intra-trial and intra-day changes. Analysis of intrasubject repeatability, both intra- and inter-day, has already been investigated in HS [80,101]. In more advanced PD stages, motor fluctuations (OFF/ON phenomena and L-Dopa-induced dyskinesia) invariably occur and lead to dissimilarity and poor repeatability in muscle synergy measurements. The introduction of a standardized protocol to manage parkinsonian motor fluctuations and achieve acceptable measurement repeatability would likely enhance the significance of muscle synergy analysis in PD. Moreover, a deeper analysis on a wider set of indices applied to motor modules and primitives, such as the short-term maximum Lyapunov exponent [58], Higuchi’s fractal dimension [102], the Hurst exponent [103], cosine similarity [35,104], R^2^ [62], full width at half maximum [44], and the centre of activity [105] could be useful in determining whether there are indices capable of discriminating parkinsonian motor symptoms with higher sensitivity than current measures. Lastly, defining comprehensive guidelines that take into account protocol design, data analysis, and the selection of sensitive indices would result in a more accurate analysis of muscle synergies in PD and lead to a better understanding of the pathophysiological basis of specific motor symptoms.

## 6. Prospects and Conclusions

Muscle synergy analysis may identify changes in PD patients, providing meaningful information about the pathophysiology of specific motor symptoms, such as locomotion and balance disorders. However, the specific CNS structures responsible for abnormal muscle synergy in PD patients remain largely unknown. Previous experimental studies in animals have demonstrated that under physiological conditions the central generators of muscle synergies are primarily located in lower-level CNS structures, such as the spinal cord and brainstem [34,51]. These lower-level structures are coordinated by higher-level regions, including the basal ganglia and cortical motor areas such as M1, the supplementary motor area, and the premotor cortex [34,51]. To date, the lack of experimental studies with muscle synergy analysis in animal models of PD makes it difficult to reach definitive conclusions about the pathophysiological role of abnormal muscle synergy in PD. 

According to the current theoretical model of functional changes in the basal ganglia circuits in PD, neurodegeneration of the substantia nigra pars compacta leads to striatal dopaminergic denervation. As a result, the output nuclei of the basal ganglia, including the substantia nigra pars reticulata and globus pallidus pars interna, increase their inhibitory projections to cortical areas and the brainstem. Accordingly, dysfunctional basal ganglia circuits lead to abnormal activation of multiple subcortical dopaminergic and non-dopaminergic structures involved in balance and locomotion, such as the mesencephalic, cerebellar, and subthalamic locomotor regions [106]. In addition to the substantia nigra pars compacta, PD is also associated with neurodegeneration of intrinsic brainstem structures, further contributing to the abnormal control of spinal generators of muscle synergy. Besides basal ganglia, cerebellum likely contributes to supraspinal modulation of muscle synergies in PD [107], according to its function in posture and gait control [108,109]. The cerebellum would play a key physiological role in the modulation of the temporal profile of muscle activation during single-joint as well as multi-joint movements [107]. Hence, the observation that L-Dopa exerts a poor effect on muscle synergies during balance and locomotion may reflect the pathophysiological involvement of non-dopaminergic pathways. This hypothesis is consistent with the clinical observation that axial motor symptoms, such as balance and gait disorders, are commonly unresponsive to L-Dopa in PD [8]. Accordingly, future studies should clarify whether alternative nonpharmacological treatments, such as those based on sensory cues [110,111,112], improve balance or gait in patients with PD by enhancing the activation of muscle synergies. For instance, in the context of gait, rhythmic patterns of auditory stimuli could lead to more synchronous motor behaviour by facilitating gait initiation [112,113,114], restoring normal patterns in gait execution [115], reducing the variability of musculoskeletal activations [116], and recruiting more consistent motor units [116] in PD patients.

In conclusion, this narrative review was designed to assess previous studies addressing muscle synergies in PD patients during specific motor tasks (e.g., balance and locomotion). Accordingly, we have first examined the theoretical background and computational methods of muscle synergies under physiological conditions. Then, we have critically discussed previous findings on this topic in PD, showing possible inconsistencies among studies. We have also speculated about the potential impact of muscle synergy analysis in providing new insights into the pathophysiology of PD motor symptoms, such as locomotion and balance disorders. Lastly, we have provided several recommendations for better designing future studies based on larger and more homogeneous cohorts of PD patients.

## Figures and Tables

**Table 1:** Demographic and clinical features of Parkinson’s disease patients in studies investigating muscle synergies.

	Sex (F/M)	Age (years)	Body Weight (kg)	Height (m)	Disease Duration (years)	Onset Side (L/R/B)	Clinical Phenotype (TD/PIGD)	H&Y	UPDRS-III		BBS	MMSE	LEDD (mg)	DBS (years)
									ON	OFF				
[87]	-	67 ± 8	80 ± 14	1.7 ± 0.1	-	-		-	-	-	-	-	-	-
[88]	2 F	66 ± 7	77 ± 9	1.7 ± 0.1	4 ± 2	-		-	37 ± 7	41 ± 10	-	-	-	-
	7 M													
[71]	6 F4 M	69 ± 6	-	-	3.5 ± 1.9	3 L		-	14 ± 10	-	-	-	412 ± 191	-
						6 R								
						2 B								
[83]	4 F 6 M	69 ± 6	80 ± 15	1.7 ± 0.1	6 ± 4	3 L 5 R 2 B		II-III	18 ± 10	27 ± 11	-	-	578 ± 144	-
[90]	10 M	61 ± 10	-	-	11 ± 5	3 L 6 R 1 B		-	27 ± 12 * 37 ± 22 **	-	-	-	715 ± 444	1.57 ± 1.2
[91]	10 M	61 ± 4	-	-	-	-		I-II	21 ± 8	32 ± 11	53 ± 5	29 ± 2	-	-
[92]	2 F	64 ± 10	-	-	7 ± 4	-	10 TD	II-III	19 ± 4	-	-	30 ± 1	423 ± 213	-
	8 M													
[93]	1 F 5 M	64 ± 17	72 ± 13	1.8 ± 0.1	7 ± 5	-	1 TD 4 PIGD 1 Undet.	I-III	30 ± 5	-	-	-	-	-

* refers to active Deep Brain Stimulation (DBS-ON); ** refers to inactive Deep Brain Stimulation (DBS-OFF); **B:** Bilateral; **BBS**: Berg Balance Scale; **DBS**: Deep Brain Stimulation; **H&Y**: Hoehn and Yahr scale; **L:** Left; **LEDD**: Levodopa Equivalent Daily Dose; **MMSE**: Mini-Mental State Examination; **PIGD:** Postural Instability/Gait Difficulty dominant; **R:** Right; **TD:** Tremor-Dominant.

**Table 2:** Experimental studies investigating muscle synergies in Parkinson’s disease.

[Ref]	Subjects	State of Therapy	Recorded Muscles	Experimental Task	Synergy Extraction	Main Findings	Conclusions	
[87]	15 PD and 14 HS	ON	Eight leg muscles bilaterally: SOL, GM, TA, VM, RF, SM, BF, GLM	10 minutes walking on a treadmill	NMF and %VAF	95% of PD require four or fewer muscle synergies, compared to 57% HS. Similar muscle weights but shifted muscle activation profile in PD. Association between walking speed and total %VAF in PD	Altered timing of modular activation may be responsible for abnormal motor control during gait in PD, rather than different muscle weighting vectors
[88]	Nine PD	ON and OFF	Eight leg muscles bilaterally: SOL, GM, TA, VM, RF, SM, BF, GLM	Overground walking and walking on a treadmill	NMF and %VAF	No differences between ON and OFF therapy for total %VAF, NoS and the muscle weighting vector. Negative correlation between total %VAF and walking speed, but no correlations with other spatiotemporal gait parameters	Dopaminergic therapy does not influence the number, structure or timing of muscle synergies
[71]	11 PD 11 HS	ON	13 leg and trunk muscle of the right side: TA, SOL, GM, GL, BF, ST, RF, VL, VM, TFL, ESL, EST, RA	Quiet standing, voluntary sway, releasing a load and fast body motion	PCA analysis with Varimax rotation and factor extraction	Four muscle synergies identified using PCA with rotation. Muscle synergies account for a lower amount of variance in PD (71.5±1.74%) than HS (78.3±1.74%). Muscle synergies are predictors of centre of pressure changes in all subjects	Organization of muscles into muscle synergies is less consistent in PD compared with HS
[83]	10 PD	ON and OFF	13 leg and trunk muscles of the right side: TA, SOL, GM, GL, BF, ST, RF, VL, VM, TFL, ESL, EST, RA	Quiet standing, voluntary sway, releasing a load and fast body motion	PCA analysis with Varimax rotation and factor extraction	Four muscle synergies identified using PCA with rotation. Muscle synergies account for a larger amount of variance in PD during ON (74.7±2.4%) than OFF (68.6±2.2%) therapy. Muscle synergies are predictors of centre of pressure changes	In PD, dopaminergic therapy makes the organization of muscles into muscle synergies more consistent during postural tasks
[90]	10 PD	ON with DBS-OFF or DBS-ON	Three leg and trunk muscles of the right side: TA, SOL, GM, GL, BF, ST, RF, VL, VM, TFL, ESL, EST, RA	Quiet standing, voluntary sway, releasing a load	PCA analysis with Varimax rotation and factor extraction	In postural tasks, four muscle synergies were identified using PCA with rotation. Muscle synergies account for similar amounts of variance in DBS-OFF (75.3±2.9%) and DBS-ON (75.1±2.9%). Muscle synergies are predictors of centre of pressure changes regardless of DBS status	DBS does not influence the organization of muscles into muscle synergies
[91]	10 PD and 10 HS	ON and OFF	six upper body muscles bilaterally: PM, DP, BB, TB, EXOB, ESL	Standing while balancing external yaw perturbation	NMF and %VAF	Higher values of total %VAF in PD than HS for NoS less than 4. Similar total %VAF during OFF and ON therapy. NoS positively correlate with MMSE scores and negatively with sub-item 3.14 of UPDRS-III (“body bradykinesia”)	PD use a lower number of muscle synergies to maintain balance. l-dopa does not influence muscle synergies during yaw postural perturbations.	
[93]	6 PD	ON	13 lower back and right leg muscle: RA, EXOB, EST, GLM, TFL, BF, VM, GM, GL, SOL, PL	Overground walking trial and standing while balancing a ramp-and-hold external perturbation before and after a rehabilitation program (three weeks of daily adapted tango classes)	NMF and %VAF	No differences in NoS after rehabilitation training. Rehabilitation improves motor module distinctness (i.e., well-defined biomechanical output between modules), consistency (reduced variability within motor modules) and generalizability (increased sharing of motor modules across gait and balance tasks)	Within- and between-module parameters (e.g., consistency, distinctness and generalizability) reflect motor performance in PD better than NoS	
[92]	10 PD and 8 HS	ON	Six right arm and upper body muscles: PM, DP, BB, TB, FR, ER	Resting tremor and reaching task with and without transcutaneous electrical stimulation of the radial nerve	NMF and %VAF	Three muscle synergies were found both in resting tremor and in reaching tasks. Cutaneous stimulation does not alter synergy vectors, but differently change the time profile of muscle synergies during resting tremor and reaching tasks	The different effects of cutaneous electrical stimulation on vector patterns and the time profile of muscle synergies may imply different spinal pathways for these signals	

**%VAF**: Variability Account For; **BB**: *biceps brachii*; **BF**: *biceps femoris*; **DBS-OFF**: inactive deep brain stimulation; **DBS-ON**: Active deep brain stimulation; **DP**: *deltoideus* – posterior portion; **ER**: *extensor carpi radialis*; **ESL**: *erector spinae* lumbar region; **EST**: *erector spinae* thoracic region; **EXOB**: external oblique; **FR**: *flexor carpi radialis*; **GL**: *gastrocnemius lateralis*; **GLM**: *gluteus medius*; **GM**: *gastrocnemius medialis*; **HS**: healthy subjects; **MMSE**: Mini-Mental State Examination; **NMF**: Non-Negative Matrix Factorization; **NoS:** Number of Synergies; **OFF:** not under dopaminergic therapy; **ON:** under dopaminergic therapy; **PCA**: Principal Component Analysis; **PD**: patients with Parkinson’s disease; **PL**: *peroneus longus*; **PM**: *pectoralis major*; **RA**: *rectus abdominis*; **RF**: *rectus femoris;* **SM**: *semimembranosus*; **SOL**: *soleus*; **ST**: *semitendinosus*; **TA**: *tibialis anterior*; **TB**: *triceps brachii*; **TFL**: *tensor fasciae latae*; **UPDRS-III:** Unified Parkinson’s Disease Rating Scale- part III; **VL**: *vastus lateralis*; **VM**: *vastus medialis*.

## Abbreviations

CNS	Central Nervous System
DBS	Deep Brain Stimulation
ELM	Extreme Learning Machine
EMG	Electromyography
H&Y	Hoehn and Yahr
HS	Healthy Subjects
ICA	Independent Component Analysis
M1	Primary Motor Cortex
NMF	Non-Negative Matrix Factorization
PCA	Principal Component Analysis
PD	Parkinson’s Disease
UPDRS	Unified Parkinson’s Disease Rating Scale
VAF	Variability accounted for
